# Surgical management of non-parasitic hepatic cyst with biliary communication: a case report

**DOI:** 10.7497/j.issn.2095-3941.2013.02.008

**Published:** 2013-06

**Authors:** Wei Cui, Hong-Yuan Zhou, Yan-Hui Zhang, Ti Zhang, Qiang Li

**Affiliations:** 1 Department of Hepatobiliary Oncology, Tianjin Medical University Cancer Institute and Hospital, Tianjin 300060, China;; 2 Department of Pathology, Tianjin Medical University Cancer Institute and Hospital, Tianjin 300060, China

**Keywords:** Hepatic cyst, biliary communication, surgical management

## Abstract

Non-parasitic hepatic cysts with biliary communication are rare. The clinical symptoms involved are not specific to this condition, thereby making diagnosis difficult and treatment controversial. Here, we report a case of 70-year-old woman complaining of abdominal satiety, combined with non-specific pain in the right upper quadrant. The abdominal contrast-enhanced MRI-scan revealed a large and thick-walled septus cystic lesion in the liver. During operation, the biliary fistula was confirmed in the cyst cavity. A silica gel tube was inserted via the cystic duct for cholangiography, which demonstrated communication between the cyst and biliary tract. We performed wide-scale cyst wall resection; the biliary fistula was completely repaired by the closure of communicated bile ducts. The postoperative course was uneventful, and the patient was discharged with no sign of cholangitis or any other symptoms. The novel surgical management via wide resection of the cyst wall and closure of biliary communication proved to be an adequate and effective procedure for treating nonparasitic hepatic cysts with biliary communication.

## Introduction

Nonparasitic hepatic cyst occurs in approximately 5% of the population, with an increasing rise in incidence with age^1^. Most of them are asymptomatic and detected incidentally by imaging examinations. In several cases, the cyst cavity is connected to the biliary system, and the preoperative diagnosis for such cases is very difficult. We reported a case of nonparasitic hepatic cyst with biliary communication, which was treated by widely cystic wall resection and closure of the bile ductile communication.

## Case report

A 70-year-old woman was admitted to the Hepatobiliary Department of Tianjin Medical University Cancer Institute and Hospital, with complaints of abdominal satiety combined with non-specific pain in the right upper quadrant, without nausea, vomit, dyspepsia, and weight loss. The patient was diagnosed to have a liver cyst for approximately 40 years, without a history of congenital cysts. Thirteen years ago, the patient was treated by percutaneous transhepatic drainage, which was guided by ultrasonography. Two years after drainage, the hepatic cyst was found to recur during the follow-up. At this time, the cyst was approximately 3 cm in size, as shown by ultrasonography. However, no treatment was administered because the cyst was asymptomatic. During the subsequent interviews, the patient recalled the obscure onset of intermittent seizures that lasted for approximately five months.

**Figure 1:**
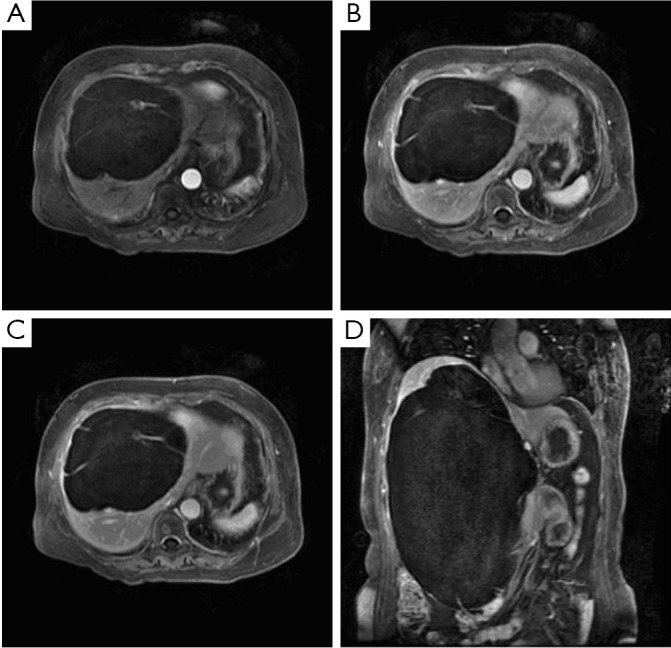
MRI-scan revealed a large cystic lesion in the liver. A. Arterial phage; B. Portal venous phage; C. Equilibrium phage; D. Coronal plane scan. A lesion of 27.9 cm × 17.9 cm × 14 cm in size occupied almost the entire liver with a thick-walled septus and cystic structure. The slight enhancement of the septum was observed after the injection of the intravenous contrast agent.

Upon physical examination, a mass was palpated in the patient’s right upper abdomen. Laboratory examination showed that the parameters of routine hematological and biochemical examinations, including tests of liver and renal function, were within normal limits. Similar results were obtained for the expression levels of tumor markers, including the blood carcinogenic embryonic antigen (CEA) and the alpha-fetoprotein (AFP). The hydatid serology test was negative. Abdominal ultrasonography identified a cyst located in the liver measuring 30 cm in diameter. The abdominal contrast-enhanced MRI-scan likewise revealed a large, thick-walled septus and cystic lesion in the liver, measuring 27.9 cm × 17.9 cm × 14 cm, which occupied almost the entire liver. A slight enhancement of the septum was present after the injection of the intravenous contrast agent. The diagnosis of cystadenocarcinoma could not be completely excluded (**Figure 1**).

Given the history of recurrence and the unpreclusive diagnosis of cystadenocarcinoma, open surgery was scheduled for the patient one week after her admission. At laparotomy, a cystic mass of approximately 30 cm in size was identified and located in the liver. Excision was conducted from the liver capsule between the cyst and the liver parenchyma. Clear fluid was drained from the cyst when the cyst wall was opened. The cystic and septus structure was observed and the bilious fluid was partially drained out. The diagnosis of hepatic cyst with biliary communication was determined based on its macroscopic appearance.

**Figure 2:**
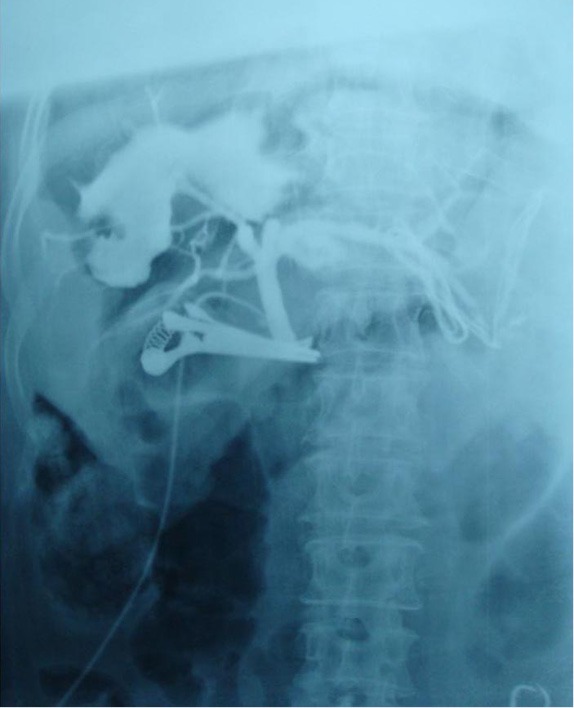
Intraoperative cholangiography shows two extravasations of the contrast medium from the branches of the hepatic bile duct.

Intraoperative cholangiography was employed to determine the existence and position of biliary fistula. A silica gel tube was inserted via the cystic duct after temporarily closing the distal end of the common bile duct with a clamp. By injecting the contrast medium (iopromide), we identified the bile ducts that communicated with the cyst. The results showed two extravasations of the contrast medium from the hepatic bile duct branches (**Figure 2**).

We then performed cystic wall resection and closed the communicated bile ducts by careful suturing. The contrast medium was subsequently reinjected, but leakage from biliary tract was no longer detected. Finally, we used omental tissue to cover the wound surface and close the abdominal cavity.

**Figure 3:**
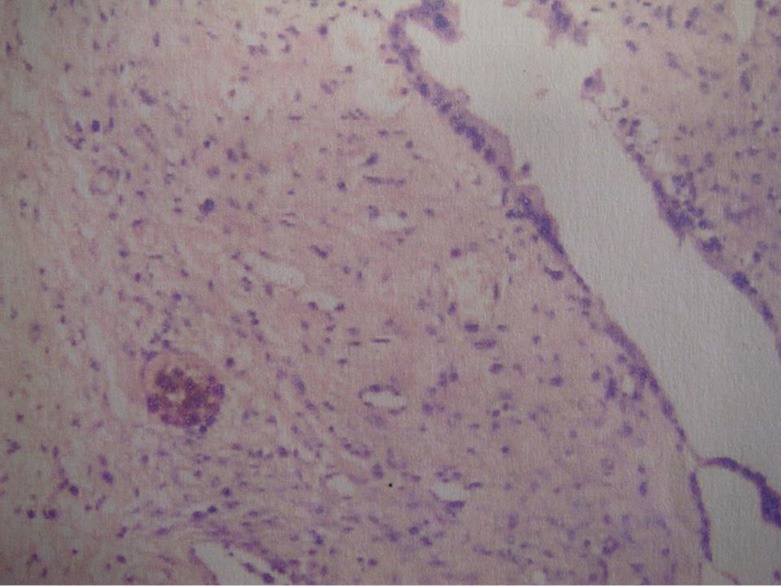
Histological pathology shows that the inner wall of the cyst is coated by epithelial tissue composed of columnar and cuboidal cells, thereby confirming the diagnosis of a liver cyst (H&E, × 200).

Pathological examination confirmed the diagnosis of a hepatic cyst (**Figure 3**). Laboratory examinations such as the routine hematological, hepatic, and renal functions were within normal limits at one week after the operation. Ultrasonography revealed no abnormalities. The patient was discharged on the 9th postoperative day with no sign of cholangitis or other symptoms.

## Discussion

Nonparasitic hepatic cysts are normally asymptomatic. These cysts are always incidental findings during laparotomy and have been recently diagnosed more frequently before operation because of the increasing use of abdominal imaging and ultrasonography. For symptomatic cases, the cysts often exhibit pressure effects, with abdominal pain as the predominant symptom^2^. For the present case, the enlarged cyst may compress the stomach and duodenum, thereby causing symptoms such as abdominal pain and satiety. Several non-parasitic hepatic cysts arose from the aberrant development of intrahepatic biliary radicals. These cysts were lined by a single layer of cuboidal or columnar epithelia, which resemble those of the bile ducts^3^. This condition may partially explain why certain hepatic cysts could communicate with the biliary tree. Cystic cases with biliary communication are mostly found in liver hydatidosis^4^. Nonparasitic hepatic cysts with biliary communication are rare, and preoperative diagnosis is very difficult. Percutaneous aspiration for inspecting the cyst contents is a direct diagnostic method to show bile communication. Jain *et al.*^5^ reported a case of draining the bilious fluid from the cyst, as guided by ultrasonography. However, although the aspirated fluid did not contain bile, the cyst could still demonstrate leakage from an intrahepatic duct. Thus, careful intraoperative examination is necessary to avoid overlooking the possible existence of biliary communication. In this case, intraoperative examination and cholangiography were performed to determine the existence and position of biliary fistula.

Several techniques such as ERCP, cholangiography, and cytography may help clinicians make diagnosis and avoid postoperative complications. Takeshi *et al.*^6^ reported a case wherein biliary communication was detected by cystography. Kilic *et al.*^7^ suggested that ERCP should be performed in asymptomatic patients suspected for biliary-cyst communication to reduce the incidence of postoperative complications.

For hepatic cysts with biliary communication, various therapeutic options have been described, including percutaneous aspiration, deroofing, cystojejunostomy, and even partial liver resection. However, uniform management has not yet to be clearly defined. The choice for therapeutic options remains debatable. The simplest method of treatment is percutaneous aspiration, which may be effective for the immediate palliation of symptoms but may invariably result in high recurrence of a cyst, particularly when the cyst exceeds 10 cm in diameter^8^. Several attempts have been made to improve this event, including the injection of sclerosing agents into the cyst. Although these methods may be more effective than aspiration alone, they have the disadvantage of irreversible sclerosing cholangtitis. This condition did not occur in the present case. Simple deroofing has also been considered a contraindication for cysts with biliary communication^9^. Missed biliary communication could prove catastrophic and may lead to biliary fistula and biliary peritonitis^10^. By contrast, liver resection is a potentially successful technique that is rarely described in the literature.

Several authors recommended cystojejunostomy as an effective surgical method for cysts with biliary communication^11^. Others argued that this procedure may increase sepsis when the previously sterile cyst becomes infected with enteric organisms after the operation^12^. For several complex cases, the procedure could lead to the relapse of cholangitis and requires repeated postoperative antibiotic treatment. Additional surgery was even needed to remove the hepatic abscess or partial hepatic tissue. Consequently, the intraperitoneal drainage, such as that via Roux-en-Y cystoenterostomy, is not advisable for this case because it may cause the formation of a postoperative biliary collection, with a considerable risk of infectious complications and additional surgical demands.

The laparoscopic approach seems preferable because it has been associated with reduced morbidity and shorter hospital stay, as compared with open surgery^13^. By contrast, several authors argued that the laparoscopic approach for the liver cyst may not offer better results in their immediate and long-term outcomes^12^. For the biliary communicating case, only Masatsugu *et al.*^14^ advocated a case that was successfully treated by laparoscopic approach. In their study, the communication was closed by interrupted sutures using a laparoscopic suturing device. However, this procedure is complex and requires more operative time and cost, as well as a highly experienced HPB surgeon. Furthermore, the choice of laparoscopic approach should be discreet. The large sizes and “difficult” locations of the cysts, such as those adjacent to the hepatic veins, were contraindications to this minimally invasive surgery. Its limitations with polycystic and relapsing cysts should also be considered^15^. Klingler *et al.*^9^ suggested that laparoscopy could be used as an exploratory approach in cases with inaccessible cysts, whereas the open technique should be employed for malignancy or biliary communication. Comparing to these procedures, the closure of the communicated bile duct under laparotomy is a more rational choice, and could be applied in most cases.

The main reason for the recurrence of the cyst is the reconstitution of the cystic wall after the operation, with the diaphragm forming a part of this wall^16^. Resecting the wide cystic wall and the secreting epithelium in the residual cystic cavity has proved important. An omental flap is likewise recognized as an ideal technique to reduce the risk of recurrence, which is contraindicated in cysts complicated by infections. Given these considerations, we performed the wide cystic wall resection and used omental tissue to cover the excisional wound and prevent recurrence. After a year of postoperative follow-up, the patient did not experience any recurrence, although she still required long-term follow-up.

In conclusion, non-parasitic hepatic cysts with biliary communication are rare, and their preoperative diagnosis is difficult. The careful detection and imaging techniques, such as cholangiography and ERCP, are useful intraoperative diagnostic techniques. Although the treatment is controversial, we advocate surgical management by wide resection of the cyst wall combined with closure of the communicated bile ducts as an effective treatment procedure. We believe that this procedure would significantly decrease the risk of infection and additional surgery and maximally avoid recurrence after the operation.
